# Meth­yl(phen­yl)phosphinic acid

**DOI:** 10.1107/S1600536811025530

**Published:** 2011-07-09

**Authors:** Robert A. Burrow, Rubia M. Siqueira da Silva

**Affiliations:** a Laboratório de Materiais Inorgânicos, Universidade Federal de Santa Maria, Av. Roraima, 1000 - Camobi, 97105-900 Santa Maria, RS, Brazil

## Abstract

The crystal structure of the title compound, C_7_H_9_O_2_P, displays O—H⋯O hydrogen bonding , which links individual mol­ecules related *via* the *c*-glide plane and translational symmetry along the crystallographic *b-*axis direction into continuous chains.

## Related literature

For background to phosphinic acids and their applications, see: Beckmann *et al.* (2009 ▶); Burrow *et al.* (2000 ▶); Burrow & Siqueira da Silva (2011 ▶); Chen & Suslick (1993 ▶); Siqueira *et al.* (2006 ▶); Vioux *et al.* (2004 ▶). For a description of the Cambridge Structural Database, see: Allen (2002 ▶) and for geometrical analysis using *Mogul*, see: Bruno *et al.* (2004 ▶).
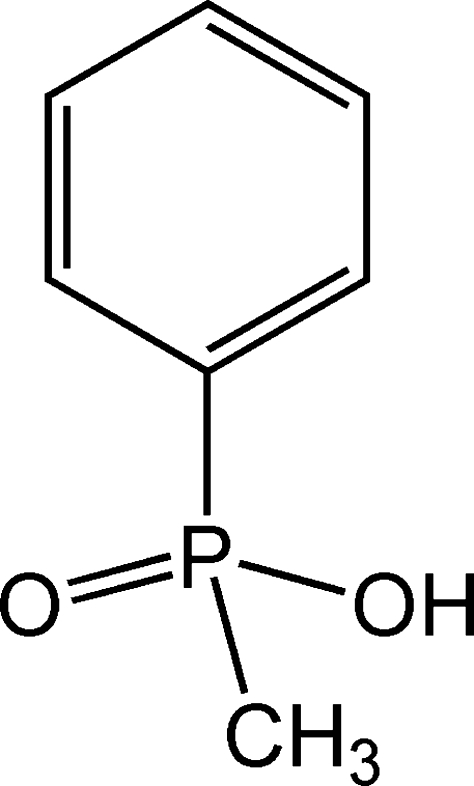


## Experimental

### 

#### Crystal data

C_7_H_9_O_2_P
*M* *_r_* = 156.11
Orthorhombic, 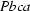

*a* = 12.4231 (8) Å
*b* = 7.8464 (5) Å
*c* = 15.9801 (10) Å
*V* = 1557.69 (17) Å^3^
*Z* = 8
Mo *K*α radiation
μ = 0.29 mm^−1^
*T* = 296 K
0.34 × 0.34 × 0.18 mm

#### Data collection

Bruker X8 Kappa APEXII diffractometer
Absorption correction: Gaussian (*SADABS*; Bruker 2009 ▶) *T*_min_ = 0.668, *T*_max_ = 0.950
19802 measured reflections
2342 independent reflections
1506 reflections with *I* > 2σ(*I*)
*R*_int_ = 0.057

#### Refinement

*R*[*F* ^2^ > 2σ(*F* ^2^)] = 0.052
*wR*(*F* ^2^) = 0.145
*S* = 1.04
2342 reflections
95 parameters
H atoms treated by a mixture of independent and constrained refinement
Δρ_max_ = 0.35 e Å^−3^
Δρ_min_ = −0.39 e Å^−3^

### 

Data collection: *APEX2* (Bruker, 2009 ▶); cell refinement: *SAINT* (Bruker, 2009 ▶); data reduction: *SAINT*; program(s) used to solve structure: *SHELXS97* (Sheldrick, 2008 ▶); program(s) used to refine structure: *SHELXL97* (Sheldrick, 2008 ▶); molecular graphics: *DIAMOND* (Brandenburg, 2008 ▶); software used to prepare material for publication: *publCIF* (Westrip, 2010 ▶).

## Supplementary Material

Crystal structure: contains datablock(s) I, global. DOI: 10.1107/S1600536811025530/zb2012sup1.cif

Structure factors: contains datablock(s) I. DOI: 10.1107/S1600536811025530/zb2012Isup2.hkl

Supplementary material file. DOI: 10.1107/S1600536811025530/zb2012Isup3.cml

Additional supplementary materials:  crystallographic information; 3D view; checkCIF report

## Figures and Tables

**Table 1:** Hydrogen-bond geometry (Å, °)

*D*—H⋯*A*	*D*—H	H⋯*A*	*D*⋯*A*	*D*—H⋯*A*
O1—H1⋯O2^i^	0.89 (3)	1.62 (3)	2.494 (2)	168 (3)

Symmetry code: (i) 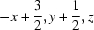.

**Fig. 1.**
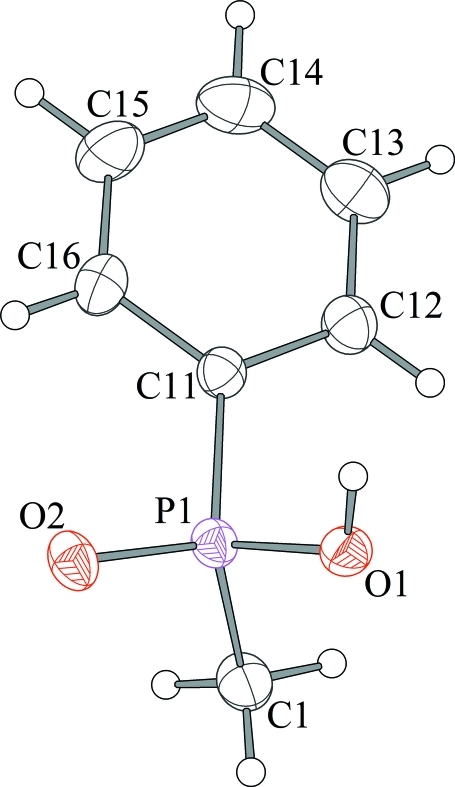
The molecular structure of (I) showing the atomic labelling scheme. The anisotropic displacement parameters are at the 30% level; H atoms are represented by circles of arbitrary size.

**Fig. 2.**
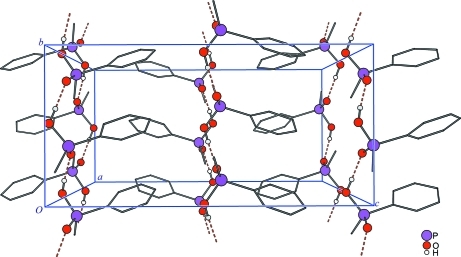
The packing diagram of (I) looking down the crystallographic a direction with the crystallographic b axis up. The H bonding are shown as red dashed lines and phenyl (C6H5) groups shown as sticks for clarity.

## supplementary crystallographic information

### Comment

Phosphinic acids have been used for the synthesis of coordination polymers [Siqueira *et al.*, 2006; Beckmann *et al.*,2009] which have the potential for a wide range of applications [Vioux *et al.*, 2004; Chen & Suslick, 1993]. As part of our ongoing research on phosphinic acids [Burrow *et al.*, 2000; Burrow & Siqueira da Silva, 2011], we report the synthesis and crystal structure of the title compound, C_7_H_9_O_2_P, (I).

The title compound, Fig. 1, crystallizes as a racemic mixture of enantiomers in the centrosymmetric space group *Pbca*. An analysis of the geometry of (I) by *Mogul* [Bruno *et al.*, 2004] using the Cambridge Structural Database [CSD, Allen, 2002] shows no unusual features; all absolute values of the *z* scores were below 1.0. An enhanced figure is provided, Fig. 2.

The crystal structure of (I) shows hydrogen bonding between the phosphinic acid moieties of the type OH···O=P—OH···O=P related by the c glide plane and translational symmetry along the crystallographic *b* direction to form continuous chains, Table 1. The very short P—O···O=P distance of 2.494 (2) Å indicates a strong hydrogen bond. This is very slightly shorter than the average O···O interaction distance in the CSD of 2.51 (5) Å for 45 observations for other phosphinic acids.

The packing diagram, Fig. 3, shows that the hydrogen bonded chains of (I) pack together in a head-to-head fashion in the crystallographic *b* direction to form columns. Neighboring columns in the crystallographic *a* direction run in the opposite direction with the neighboring methyl groups packing together. The effect creates a pseudo-lamellar structure parallel to the crystallographic *ab* plane where the phosphinate groups and methyl groups are in a plane surrounded by phenyl groups on either side. There are no phenyl-phenyl interactions. The distance between layers is half the *c* axis distance, 7.9900 (5) Å.

### Experimental

To a solution of phenylphosphinic acid (2.0 g, 14.1 mmol) in dichloromethane, 30 ml diisopropylethylamine (5.16 ml, 29.6 mmol) and trimethylsilyl chloride (3.74 ml, 29.6 mmol) were separately added at 0 °C under argon. The reaction mixture was stirred at room temperature for 2–3 h, cooled to 0 °C and iodomethane (0.97 ml, 15.6 mmol) was added. After further stirring at room temperature for 24 h, the solvent was removed under vacuum. The residue was suspended in hydrochloric acid (2 *M*, 20 ml) and filtered on a glass frit under vacuum. The white solid was washed with acetone and dried giving a yield of 1.60 g (66%) of pure product. Crystals were obtained by slow evaporation from a methanol solution. IR (KBr): 1439 (*s*), 1304 (w), 1266 (*m*), 1171 (*s*), 1134 (*s*), 1049 (m, br), 1026 (*m*), 982 (*versus*), 881 (*s*), 779 (*s*), 745 (*s*), 700 (*m*), 512 (*m*), 482 (*m*), 439 (w) cm^-1^. C_7_H_9_O_2_P (156.12): calc.: C 53.85, H 5.81; found: C 52.77, H 6.01%.

### Refinement

The H atom on O1 was found in the difference Fourier map and its position was allowed to refine freely while its isotropic displacement parameter was set to 1.5 times *U*_eq_ of O1. H atoms were positioned geometrically and allowed to ride on their parent atoms with C—H bond lengths of 0.93 Å (aromatic CH) and 0.96 Å (methyl CH_3_) and isotropic displacement parameters equal to 1.2 times *U*_eq_ of the parent atom.

### Crystal data

**Table:**

C_7_H_9_O_2_P	*D*_x_ = 1.331 Mg m^−^^3^
*M**_r_* = 156.11	Melting point = 402–408 K
Orthorhombic, *P**b**c**a*	Mo *K*α radiation, λ = 0.71073 Å
*a* = 12.4231 (8) Å	Cell parameters from 788 reflections
*b* = 7.8464 (5) Å	θ = 2.5–22.7°
*c* = 15.9801 (10) Å	µ = 0.29 mm^−^^1^
*V* = 1557.69 (17) Å^3^	*T* = 296 K
*Z* = 8	Irregular block, colourless
*F*(000) = 656	0.34 × 0.34 × 0.18 mm

### Data collection

**Table:**

Bruker X8 Kappa APEXII diffractometer	2342 independent reflections
Radiation source: fine focus ceramic X-ray tube	1506 reflections with *I* > 2σ(*I*)
graphite	*R*_int_ = 0.057
Detector resolution: 8.3333 pixels mm^-1^	θ_max_ = 30.5°, θ_min_ = 3.0°
0.5° φ and ω scans	*h* = −17→16
Absorption correction: gaussian (*SADABS*; Bruker 2009)	*k* = −11→11
*T*_min_ = 0.668, *T*_max_ = 0.950	*l* = −22→22
19802 measured reflections	

### Refinement

**Table:**

Refinement on *F*^2^	Primary atom site location: structure-invariant direct methods
Least-squares matrix: full	Secondary atom site location: difference Fourier map
*R*[*F*^2^ > 2σ(*F*^2^)] = 0.052	Hydrogen site location: inferred from neighbouring sites
*wR*(*F*^2^) = 0.145	H atoms treated by a mixture of independent and constrained refinement
*S* = 1.04	*w* = 1/[σ^2^(*F*_o_^2^) + (0.0622*P*)^2^ + 0.898*P*] where *P* = (*F*_o_^2^ + 2*F*_c_^2^)/3
2342 reflections	(Δ/σ)_max_ < 0.001
95 parameters	Δρ_max_ = 0.35 e Å^−^^3^
0 restraints	Δρ_min_ = −0.39 e Å^−^^3^

### Special details

**Table:**

Geometry. All e.s.d.'s (except the e.s.d. in the dihedral angle between two l.s. planes) are estimated using the full covariance matrix. The cell e.s.d.'s are taken into account individually in the estimation of e.s.d.'s in distances, angles and torsion angles; correlations between e.s.d.'s in cell parameters are only used when they are defined by crystal symmetry. An approximate (isotropic) treatment of cell e.s.d.'s is used for estimating e.s.d.'s involving l.s. planes.
Refinement. Refinement of *F*^2^ against ALL reflections. The weighted *R*-factor *wR* and goodness of fit *S* are based on *F*^2^, conventional *R*-factors *R* are based on *F*, with *F* set to zero for negative *F*^2^. The threshold expression of *F*^2^ > σ(*F*^2^) is used only for calculating *R*-factors(gt) *etc*. and is not relevant to the choice of reflections for refinement. *R*-factors based on *F*^2^ are statistically about twice as large as those based on *F*, and *R*- factors based on ALL data will be even larger.

### Fractional atomic coordinates and isotropic or equivalent isotropic displacement parameters (Å^2^)

**Table:**

	*x*	*y*	*z*	*U*_iso_*/*U*_eq_	
P1	0.82414 (4)	0.86402 (6)	0.46093 (3)	0.03532 (18)	
O1	0.85676 (12)	1.0170 (2)	0.51708 (10)	0.0413 (4)	
H1	0.823 (2)	1.114 (4)	0.504 (2)	0.062*	
O2	0.71118 (12)	0.8030 (2)	0.47403 (10)	0.0456 (4)	
C1	0.9197 (2)	0.7033 (2)	0.48579 (17)	0.0505 (5)	
H1A	0.9154	0.6771	0.5444	0.076*	
H1B	0.9907	0.7433	0.4727	0.076*	
H1C	0.9044	0.6026	0.4537	0.076*	
C11	0.84247 (18)	0.9303 (2)	0.35447 (12)	0.0384 (5)	
C12	0.9378 (2)	1.0107 (2)	0.32936 (16)	0.0503 (5)	
H12	0.9933	1.0267	0.3676	0.06*	
C13	0.9496 (2)	1.0661 (4)	0.24804 (17)	0.0626 (7)	
H13	1.0128	1.1201	0.2317	0.075*	
C14	0.8681 (2)	1.0420 (4)	0.19095 (17)	0.0684 (8)	
H14	0.8762	1.0804	0.1363	0.082*	
C15	0.7754 (2)	0.9616 (4)	0.21438 (17)	0.0735 (9)	
H15	0.7211	0.9447	0.1752	0.088*	
C16	0.7612 (2)	0.9050 (2)	0.29563 (17)	0.0559 (7)	
H16	0.6977	0.8504	0.3109	0.067*	

### Atomic displacement parameters (Å^2^)

**Table:**

	*U*^11^	*U*^22^	*U*^33^	*U*^12^	*U*^13^	*U*^23^
P1	0.0314 (2)	0.0321 (2)	0.0425 (2)	−0.0001 (2)	0.0008 (2)	0.0015 (2)
O1	0.0458 (9)	0.0356 (8)	0.0426 (9)	0.0015 (7)	−0.0052 (7)	0.0003 (7)
O2	0.0328 (8)	0.0381 (8)	0.0660 (11)	−0.0008 (7)	0.0059 (7)	0.0031 (7)
C1	0.0406 (13)	0.0406 (13)	0.0703 (16)	0.0049 (10)	−0.0013 (11)	0.0057 (11)
C11	0.0404 (11)	0.0356 (11)	0.0391 (11)	−0.0005 (9)	−0.0005 (9)	−0.0028 (9)
C12	0.0444 (13)	0.0555 (15)	0.0511 (14)	−0.0032 (11)	0.0031 (11)	0.0012 (11)
C13	0.0661 (18)	0.0662 (17)	0.0555 (16)	−0.0018 (15)	0.0193 (14)	0.0039 (14)
C14	0.100 (3)	0.0658 (18)	0.0395 (14)	0.0060 (18)	0.0101 (15)	0.0029 (13)
C15	0.095 (2)	0.078 (2)	0.0473 (16)	−0.007 (2)	−0.0204 (15)	−0.0019 (15)
C16	0.0537 (16)	0.0614 (16)	0.0525 (14)	−0.0097 (13)	−0.0108 (11)	−0.0034 (11)

### Geometric parameters (Å, °)

**Table:**

P1—O2	1.4976 (16)	C12—C13	1.378 (4)
P1—O1	1.5526 (16)	C12—H12	0.93
P1—C1	1.777 (2)	C13—C14	1.375 (4)
P1—C11	1.794 (2)	C13—H13	0.93
O1—H1	0.89 (3)	C14—C15	1.366 (5)
C1—H1A	0.96	C14—H14	0.93
C1—H1B	0.96	C15—C16	1.383 (4)
C1—H1C	0.96	C15—H15	0.93
C11—C16	1.394 (3)	C16—H16	0.93
C11—C12	1.401 (3)		
			
O2—P1—O1	114.29 (9)	C13—C12—C11	120.1 (2)
O2—P1—C1	111.56 (10)	C13—C12—H12	119.9
O1—P1—C1	104.21 (11)	C11—C12—H12	119.9
O2—P1—C11	110.13 (10)	C14—C13—C12	120.3 (3)
O1—P1—C11	106.92 (10)	C14—C13—H13	119.9
C1—P1—C11	109.45 (11)	C12—C13—H13	119.9
P1—O1—H1	114.(2)	C15—C14—C13	120.1 (3)
P1—C1—H1A	109.5	C15—C14—H14	119.9
P1—C1—H1B	109.5	C13—C14—H14	119.9
H1A—C1—H1B	109.5	C14—C15—C16	120.9 (3)
P1—C1—H1C	109.5	C14—C15—H15	119.6
H1A—C1—H1C	109.5	C16—C15—H15	119.6
H1B—C1—H1C	109.5	C15—C16—C11	119.7 (3)
C16—C11—C12	118.9 (2)	C15—C16—H16	120.2
C16—C11—P1	120.4 (2)	C11—C16—H16	120.2
C12—C11—P1	120.63 (17)		
			
O2—P1—C11—C16	−6.3 (2)	P1—C11—C12—C13	−177.8 (2)
O1—P1—C11—C16	−131.0 (2)	C11—C12—C13—C14	−0.4 (4)
C1—P1—C11—C16	116.7 (2)	C12—C13—C14—C15	−0.5 (5)
O2—P1—C11—C12	172.62 (18)	C13—C14—C15—C16	0.7 (5)
O1—P1—C11—C12	47.9 (2)	C14—C15—C16—C11	0.0 (5)
C1—P1—C11—C12	−64.4 (2)	C12—C11—C16—C15	−0.9 (4)
C16—C11—C12—C13	1.1 (4)	P1—C11—C16—C15	178.0 (2)

### Hydrogen-bond geometry (Å, °)

**Table:**

*D*—H···*A*	*D*—H	H···*A*	*D*···*A*	*D*—H···*A*
O1—H1···O2^i^	0.89 (3)	1.62 (3)	2.494 (2)	168 (3)

Symmetry codes: (i) −*x*+3/2, *y*+1/2, *z*.
